# Fun(gi)omics: Advanced and Diverse Technologies to Explore Emerging Fungal Pathogens and Define Mechanisms of Antifungal Resistance

**DOI:** 10.1128/mBio.01020-20

**Published:** 2020-10-06

**Authors:** B. Ball, M. Langille, J. Geddes-McAlister

**Affiliations:** a Department of Molecular and Cellular Biology, University of Guelph, Guelph, Ontario, Canada; University of Texas Health Science Center at Houston

**Keywords:** omics technologies, emerging fungal pathogens, fungal pathogenesis, antifungal resistance, data integration, genomics, metabolomics, multi-omics, proteomics, transcriptomics

## Abstract

The landscape of infectious fungal agents includes previously unidentified or rare pathogens with the potential to cause unprecedented casualties in biodiversity, food security, and human health. The influences of human activity, including the crisis of climate change, along with globalized transport, are underlying factors shaping fungal adaptation to increased temperature and expanded geographical regions. Furthermore, the emergence of novel antifungal-resistant strains linked to excessive use of antifungals (in the clinic) and fungicides (in the field) offers an additional challenge to protect major crop staples and control dangerous fungal outbreaks. Hence, the alarming frequency of fungal infections in medical and agricultural settings requires effective research to understand the virulent nature of fungal pathogens and improve the outcome of infection in susceptible hosts. Mycology-driven research has benefited from a contemporary and unified approach of omics technology, deepening the biological, biochemical, and biophysical understanding of these emerging fungal pathogens. Here, we review the current state-of-the-art multi-omics technologies, explore the power of data integration strategies, and highlight discovery-based revelations of globally important and taxonomically diverse fungal pathogens. This information provides new insight for emerging pathogens through an in-depth understanding of well-characterized fungi and provides alternative therapeutic strategies defined through novel findings of virulence, adaptation, and resistance.

## Abstract (precis)

The landscape of infectious fungal agents includes previously unidentified or rare pathogens with the potential to cause unprecedented casualties in biodiversity, food security, and human health. The influences of human activity, including the crisis of climate change, along with globalized transport, are underlying factors shaping fungal adaptation to increased temperature and expanded geographical regions. Furthermore, the emergence of novel antifungal-resistant strains linked to excessive use of antifungals (in the clinic) and fungicides (in the field) offers an additional challenge to protect major crop staples and control dangerous fungal outbreaks.

## INTRODUCTION

Emerging fungal diseases have recently entered center stage to become notorious pathogens for wreaking havoc on plants, animals, and humans. For plants, the threat of fungal disease is a longstanding economic burden on essential food crops. For instance, the fungal-like oomycete Phytophthora infestans devasted potato crops in the 1840s, igniting the Irish potato famine, and modern-day agricultural downfalls repeatedly occur from wheat- and rice-targeting fungi, including Magnaporthe oryzae, Botrytis cinerea, and Fusarium graminearum (1–3). Epidemic outbreaks of fungal and oomycete infections of food crops have accumulated a loss of global feeding capacity by 8.5% (approximately 650 million people) (4). Such historical fungal threats to agriculture are correlated with crop domestication; however, the role of fungi as human and animal pathogens is a relatively recent discovery (5). This underrecognition is surprising due to the commonality of superficial fungal infections, meaning hair, skin, and nails, apparent in more than 1 billion people globally (6). Furthermore, invasive fungal infections capable of systemic disease in humans are in the spotlight due to unacceptably high mortality rates, most commonly reported in individuals with immunodeficient predispositions, and estimated to cause more than 1.6 million deaths annually (7). This scenario is exacerbated by the limited repertoire of antifungal therapeutics and the absence of available vaccines, as well as challenges ranging from a lack of compulsory endemic reporting to deficits in research and development funding (8–10, 129).

The rising occurrence of infectious fungal agents features many species causing disease, which are commonly equipped with biological advantages over the host. For example, rapid reproductive capabilities, resilient dispersal cycles, and genomic plasticity featuring horizontal gene transfer as well as hybridization and recombination, expedite the evolution of adaptation (4, 11). In addition, the emergence of new fungal pathogens, commonly accelerated with the influence of human activity, including rising global temperatures and globalized transport, leads to the unnatural dispersal of fungi. These events have abetted the exposure of previously unidentified or rare fungal species and facilitated the establishment of formerly uncolonized and unsuitable regions (12–14). For instance, Cryptococcus gattii, commonly located in tropical and subtropical regions, caused an outbreak in the Pacific Northwest featuring advanced virulence against healthy hosts and has continually spread across Western North America (15, 16). Furthermore, the global mycobiome has shifted in recent decades due to the dual use of medically approved antifungals as agricultural fungicides (17, 18). The consequences of these actions supported the emergence of novel antifungal-resistant strains, including azole-resistant Aspergillus fumigatus and a recent epidemiological increase in drug-resistant invasive candidiasis inflicted by non-*albicans* species (19, 20). Additionally, a novel *Candida* pathogen, Candida auris, frequently associated with hospital-acquired infections and multidrug resistance (MDR), was initially discovered in Japan in 2009 and has now migrated worldwide (21). The mysterious circumstances of C. auris simultaneous emergence on three different continents may be the first representation of the consequences of rising global temperatures (22, 23).

**FIG 1:**
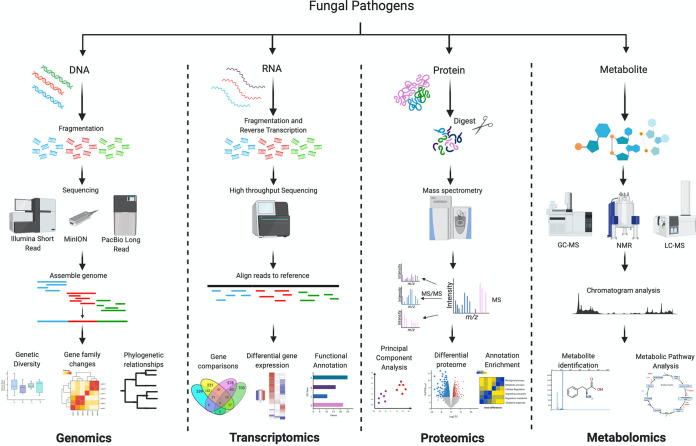
Outline of omics technologies highlighted in this review. Each technology provides in-depth profiling at the desired molecular level through the application of advanced genomic (genes), transcriptomic (transcripts), proteomic (proteins), and metabolomic (metabolites) platforms. The steps pertinent to each technology are presented along with critical components for data analysis and interpretation.

To combat the fungal threat to global health and food security, as well as relieve the future burden on health care systems, detailed analyses are necessary to elucidate the molecular mechanisms of host susceptibility and fungal pathogenesis. Recent technological advancements have brought researchers into the “omics” era, contributing an innovative toolbox of genomics, transcriptomics, proteomics, and metabolomics (Fig. 1). The innovation of omics technology combined with systems biology, bioinformatics, and high computational power captures a contemporary perspective on understanding the complexities of fungal disease, including the processes of virulence, host adaptation, and future druggable targets (24). Each level of the omics platform provides specific insights into the studied pathogen; for example, advances in next-generation sequencing (NGS) accurately diagnose often misidentified fungal pathogens (e.g., C. auris and the closely related species Candida haemulonii) (25). Moreover, the integration of multiple omics approaches for the study of emerging fungal pathogens contains the powerful potential to discover systems-scale architecture of fungal pathogenesis rapidly, and it permits a deeper understanding of the interplay between molecular levels controlling such pathogenesis. The purpose of this review is to recognize the current systems biology investigations and findings of well-characterized and emerging fungal pathogens to provide suggestions for new applications of omics platforms to inform a global initiative to combat the ever-increasing rates of antifungal resistance in both medical and agricultural settings.

## GENOMICS

The emergence of new fungal pathogens has unfolded in parallel with the genomic sequencing revolution, providing tools to help broaden the evolutionary knowledge of virulence, host range, and geographic expansion of these pathogens. Current approaches for genomic analysis include assembling a genome *de novo* for a novel species and resequencing using an exclusive reference genome to identify variants through comparisons (26–28). Multiple methods for assembly and resequencing are available, including short-read sequence technology (e.g., Illumina) and long-read technology beneficial for a repetitive genome (e.g., Pacific Biosciences and Oxford Nanopore) (29). By capitalizing on such advancements in genomics technology, crucial information about the genome composition of rapidly emerging fungal species reveals gene family expansions and gene loss events, which outlines their functional potential for virulence and host infection.

### *De novo* sequencing for genome assembly.

For *de novo* sequencing, the availability of inexpensive, high-throughput whole-genome sequencing enables assemblies for a wider variety of fungal pathogens, including newly emerged and rarely observed species (30–32). For example, *Candida* spp. are a prominent cause of fungal infections in immunocompromised patients, with a high hospital-acquired occurrence (18, 33). Historically, the common causative agent of candidemia is Candida albicans; however, the clinical epidemiology has shifted to include a growing list of emergent and rare species (e.g., C. auris, Candida glabrata, and Candida inconspicua) (34, 35). The type strain of C. inconspicua was *de novo* assembled, followed by the sequencing of 10 additional isolates, all originating from regions of endemicity within European countries (36). Analysis of the variants achieved an improved understanding of the recent pathogenic evolution of C. inconspicua and indicated a highly heterozygous genome with interspersed events of loss of heterozygosity. The isolates’ phylogenetic relationship via single nucleotide polymorphism (SNP) reconstruction revealed two distinct clades not correlated with geographical distribution. This study proposed a hybrid nature of the C. inconspicua lineage, raising concern for this medical pathogen’s plasticity and high propensity for antifungal resistance. In another example, preliminary genomic studies of C. auris isolates revealed the simultaneous emergence of four clades in distinct geographic regions (22). To close knowledge gaps of C. auris’ unique evolution, a comparative genomic analysis was achieved with the addition of highly complete whole-genome assemblies from isolates within each of the four clades as well as closely related species within the *C. haemulonii* clade (37). Phylogenomic and gene family changes in C. auris (relative to related species) revealed gene expansions contributing to antifungal resistance and virulence shared across the C. auris clades, including species-specific expansion of oligopeptide transporters, siderophore iron transporters, and secreted lipases. Furthermore, azole resistance in the MDR C. auris isolates corresponded to increased copy number and mutations of the *ERG11* gene (38). The association between *ERG11* point mutations and azole resistance is supported by a recent study identifying multiple Erg11 amino acid substitutions in clinical isolates across two C. auris clades. Interestingly, reduced azole susceptibility was measured in Saccharomyces cerevisiae containing a heterologous expression system of C. auris *ERG11* alleles encoding either the Y132F or K143R amino acid difference; this relationship offers value as potential resistant molecular markers for clade specific isolates (39).

### Gene expansions and duplications.

Comparative whole-genome analysis between the variation of clinical isolates to environmental or nonpathogenic isolates highlights an avenue to distinguish expansions and duplications of protein families for niche adaptation. For example, Fusarium oxysporum has a widely distributed species complex (FOSC) encompassing both soilborne and clinical pathogens (40). Two F. oxysporum human isolates featuring adverse clinical manifestations were compared against a distantly related tomato wilt pathogenic species. An invasive fusariosis isolate, capable of causing opportunistic invasive infections, was sequenced using a whole-genome shotgun approach. To confirm confidence in the genome assembly and supplement annotation, RNA sequencing (RNA-Seq) was applied, resulting in 99.8% alignment of transcripts (41). From these data, four unique lineage-specific chromosomes were exclusively reported and showed homology to an isolate associated with the 2005–2006 *Fusarium* keratitis outbreak (42, 43). These lineage-specific chromosomes revealed enrichment in virulence-associated genes necessary for overcoming nutritional immunity (e.g., metal ion and cation transporters). Additionally, homologs present in both strains include expansions of the virulence-associated alkaline pH-responsive transcription factor PacC/Rim1p as well as a homolog of ceruloplasmin, a major mammalian copper-carrying protein in the blood. These results associate the transposon-rich lineage-specific chromosomes as a focal point in two human-pathogenic fungal genomes for host adaptation and pathogenicity for establishing human infection. Interestingly, phytopathogenic *Fusarium* species lacked any homologous sequences to ceruloplasmin, whereas orthologs were identified in other opportunistic human fungal pathogens, including the black yeast Exophiala oligosperma. The ability to access and circumvent the bioavailability of metals from the host is an essential virulence factor in human-pathogenic fungi. This study offers primary evidence connecting fungal accessory chromosomes as transposon-rich hot spots to promote fungal adaptation of human hosts.

### Identification of gene markers.

The use of available genome sequences and improved reference assemblies promotes a comparison of sequences of isolates representing populations or clinical outbreaks to identify conserved gene markers. For example, a number of agricultural crops are infected with multiple species of *Fusarium* to cause wilt and root rot disease, which demands diagnostic tools to discriminate the polyphyletic nature of Fusarium oxysporum *formae speciales* (44, 45). Such a polyphyletic nature prevents molecular identification of novel strains; therefore, an alternative detection method was developed by comparing 88 whole-genome sequences of cucurbit-infecting F. oxysporum strains to identify multiple effector genes as putative markers (46). This approach uniquely targeted virulence-related genes, providing high specificity for hard-to-distinguish closely related *forma specialis.* Another application in the medical sector developed a robust pipeline using an NGS-based amplicon of internal transcribe spacer 1 (ITS1) to advance sequencing strategies of fungal community representatives of the lower respiratory tract during infection (47). This barcoding analysis was explored against the mycobiome of bronchoalveolar lavage (BAL) specimens obtained from the lower respiratory tracts with known *Blastomyces* culture status. It resulted in 91.4% accuracy for the identification of *Blastomyces* as well as unbiased profiling of other present fungal taxons to define novel gene markers (47). These studies demonstrate robust gene marker technologies to offer an alternative opportunity for identification of hard-to-distinguish novel fungal species by using an unbiased culture-independent strategy. Furthermore, the adoption of shotgun metagenomic technology for disease diagnostics can permit unbiased sequencing of polymicrobial communities isolated directly from clinical samples, with powerful prediction capabilities of antimicrobial resistance, virulent gene identification, and high resolution if performing molecular typing (48). For instance, a metagenomic diagnostic-based approach of fungal infections of the central nervous system (CNS), such as C. gattii, would provide relief in medical settings with limited diagnostic capabilities for the high diversity of etiological agents capable of CNS infections (49).

### Databases and bioinformatics pipelines.

Lastly, exploiting the vast collection of genomic databases offers an alternative approach to mapping the genetic diversity of virulent and antifungal-resistant isolates. A public bioinformatics pipeline was recently generated to characterize novel strains within the azole-resistant A. fumigatus metapopulation (50). A. fumigatus is a globally distributed opportunistic pathogen with mortality rates of 40% to 90% in immunocompromised cases; infection severity is influenced by increasing resistance to azole drug therapy stemming from the large-scale dual azole use for crop protection (51, 52). In this study, the bioinformatics platform originated from hierarchical clustering and multivariate analysis of the STRA*f* collection of 4,049 A. fumigatus isolates genotyped at nine microsatellites within the bounds of two known resistant genotypes to reveal the clustering of isolates into two broadly defined populations with resistance mechanisms (50). With this information, the authors characterized novel fungal strains and provide a framework for establishing similar pipelines in other fungal species. As antifungal resistance and emerging species within the health and agricultural industries continue to increase, the unprecedented scale of genomics promises to uncover new modes of virulence and mechanisms of resistance through integration of multiple fungal genome sequences.

## TRANSCRIPTOMICS

Transcriptome analyses offered the first attempt to capture the fingerprint of diverse gene expression within a cell at a specific moment in time. Transcript profiling has evolved from reverse transcriptase quantitative PCR (RT-qPCR) to the incorporation of genomic-scale approaches, including serial analysis of gene expression (SAGE) and DNA microarrays that measure the abundance of a predefined transcript pools (53–55). Presently, the dominant method for transcriptome analysis is RNA-Seq, this state-of-the-art technique combines high-throughput sequencing and computational bioinformatics to capture and sequence transcripts of cDNA and accurately derive relative gene expression levels (56). Deep sampling of the transcriptome provides a platform to understand the intricate regulatory networks involved during infection that are controlled by developmental stages, environmental conditions, and nutrient availability.

### Virulence factor determination.

The integration of a genome-wide analysis with targeted transcriptomics is a robust endeavor to elucidate virulence factors in understudied pathogens. *Scedosporium* spp. are ubiquitous environmental fungi and, until recently, neglected pathogens that emerged as colonizers of the respiratory tract with high incidence in individuals with cystic fibrosis (CF) (57, 58). Recently, the sequenced genome of Scedosporium apiospermum facilitated a bioinformatics mining analysis using A. fumigatus iron-related proteins as the query to reveal orthologs of S. apiospermum genes putatively involved in iron metabolism (59). Targeted transcriptomics using RT-qPCR of conidia growth under iron excess or deprivation conditions validated the expression of several iron-regulating genes, including intra/extracellular siderophore biosynthesis genes, ferrisiderophore transport genes, reductive iron assimilation genes (i.e., ferric reductase, multicopper ferroxidase, and iron permease), and a vacuolar iron importer gene (59). Transcriptome verification of iron acquisition in S. apiospermum revealed a critical virulence factor for establishing infection within the unique iron-rich environment of a CF lung.

### Dynamic interactions of host-pathogen interface.

To uncover novel targeted therapeutic strategies, it is essential to understand the dynamic interactions at the host-pathogen interface. Such an approach is possible by assessing expression levels during the colonization and dissemination of an invading fungus and the opposing response of the host. The molecular feedback triggered by the host cell upon infection offers vital information into the pathogenesis, invasion, and virulence of the pathogenic fungi. For instance, the response of alveolar epithelial cells to Scedosporium aurantiacum infection was studied using RNA-Seq Illumina with paired-end sequence reads to reveal 3,950 differentially expressed genes (60). Infected host alveolar cells exhibited 2,008 upregulated genes with trends in cell death, inflammation, wound healing, and cell repair. Notably, *MUC5*, a gene involved in mucin production, had higher expression levels during infection, indicating a possible clearance response of the alveolar epithelial cells. Gene network analysis designated a strong upregulation in the inflammatory NF-κB pathway and proinflammatory cytokines CXCL8/interleukin (IL)-8 and IL-11. The NF-κB inflammation pathway mediates IL-8 production during infection of human respiratory epithelial cells via other filamentous lung pathogens such as A. fumigatus (61, 62). The transcriptome host profile suggests the ability of lung epithelial cells to provide multipurpose protection from inhaled fungal pathogens, including a physical barrier, as well as recognize germinating conidia followed by the initiation of a programmed defense response.

The interplay of infection from the invasive white mold fungus Sclerotinia sclerotiorum in Arabidopsis thaliana was also defined at the transcriptional level to examine gene regulation within prime infection regions (63). Invasive hyphae of *S. sclerotiorum* intracellularly colonize host tissues while host cell death and tissue maceration originate at the center of the site of initial infection. This difficult-to-control fungus notoriously devastates global food security and recently spread to previously uninhabited geographical niches (64–67). The global infection transcriptome was investigated based on the spatial organization of invasive hyphae relative to that of the central mycelium cells. Transcriptional responses highlighted area-specific reprogramming in apex cells compared to transcription in central cells for 135 and 197 induced genes, respectively. The functions enhanced between the two infection regions include fungal toxin biosynthesis at the apex and essential metabolic process required for plant-host colonization at the mycelium center. The nature of these combined functions resolves the multicellular communication during host colonization to generate a pattern for specific division-of-labor genes in *S. sclerotiorum* invasive hyphae. This study also features powerful systems biology and modeling approaches using a genome-scale metabolic model to demonstrate how hostile host environments, in contact with the fungal pathogen, direct the local transcriptional programming to favor multicellular hyphal production for cooperative growth.

The yeast-fungal pathogen Cryptococcus neoformans adapts to various hostile environments during its infection cycle ranging from intracellular replication within macrophages to dissemination within the cerebral spinal fluid (CSF) for manifestation of cryptococcal meningitis or meningoencephalitis (68). Using RNA-Seq technology, different biologically relevant stresses promoting infection were assessed in understudied clinical and environmental isolates from C. neoformans VNI and VNB lineages (69). This study highlighted lineage-specific differentially expressed genes clustered within the genome and enriched in subtelomeric regions, suggesting the potential for higher selection pressure. Gene expression in different pathogenic stages of cryptococcal meningitis was assessed using a subarachnoid space and an intracellular macrophage model to highlight metabolic and stress-adaptive capabilities. Expressed genes involved in stress responses, inositol phosphate metabolism, and lipid metabolism were identified (69), defining the relationship between the host and pathogen at the transcript level, with the capacity for a transferable analysis to the closely related novel C. gattii.

### Stress responses and survival.

Biological responses to stress often dictate the survival state of pathogenic fungi’s responsiveness to antimicrobial agents. Recently, a study identified different molecular pathways and how fluconazole resistance differs in response to acidic pH niches in biofilms of C. glabrata. C. glabrata is an emerging threat in severely immunocompromised individuals and is associated with a higher mortality rate than other non-*albicans* spp. capable of causing candidemia, a nosocomial bloodstream infection (70). Moreover, the difficulty of treatment increases due to its ability to adhere and form biofilms on host tissues and medical devices, heightening its intrinsic resistance to antifungal drugs. Illumina whole-transcriptome sequencing identified 4,213 upregulated genes in C. glabrata biofilms supplemented with acetate and fluconazole (71). Enrichment analysis revealed induced gene expression in DNA replication and ergosterol and ubiquinone biosynthesis processes. Notably, this study suggests that the carbon source dictates the degree to which fluconazole induces ergosterol-related genes. In another study, *de novo* transcriptome assembly of C. auris biofilm cells generated over various time points was performed, and sequenced sample reads were assembled into an ∼11.5-Mb transcriptome consisting of 5,848 genes (72). The mature stages of biofilm development indicated several enriched genes encoding efflux pumps and major facilitator superfamily transporters. *Candida* biofilms, concerningly, have reported resistance to three classes of antifungals; therefore, these temporal transcriptome analyses provide substantial new insight into mechanisms of antifungal resistance.

## PROTEOMICS

Mycology-focused research has profoundly progressed over the last 2 decades due to the impressive technological advancement of mass spectrometry (MS)-based proteomics (73). Proteomics profiles the broad view of gene expression patterns, including quantification of protein production, posttranslational modifications (PTMs), alternate protein isoforms, and interaction networks (74). The use of high-resolution MS is a powerful tool to derive distinct proteomic applications, including targeted proteomics that quantifies specific proteins of interest and top-down proteomics, which introduces whole intact proteins to be measured and is highly sensitive to proteoform differentiation. A popular technique featured for studying neglected fungal species is the unbiased discovery-driven platform of bottom-up proteomics, which subjects the proteins to enzymatic digestion prior to MS analysis (75–78). These distinct MS-based applications are capable of profiling from supernatants, cells, tissues, and organs to provide comprehensive information about fungal pathogenesis, including host-fungal interactions, virulence attributes, and biomarker discovery.

### Regulation of signal transduction pathways.

Quantitative proteomic investigations are not restricted to the site of protein production; thus, comprehensive profiling of a fungal system can evolve around distinct cellular compartments (i.e., the “cellular proteome”), the extracellular environment (i.e., the “secretome”), and alternative fungal survival morphogenic states (73). This dynamic nature of assessing protein production is valuable for the analysis of fungal signal transduction pathways that have global impacts across the total proteome and secretome. For example, in C. neoformans, the cyclic-AMP/protein kinase A (PKA) pathway is a key player in the expression of critical virulence factors necessary for infection (79). The Pka1-modulated proteome of C. neoformans was quantitatively profiled to reveal the influence of Pka1 regulation on functions such as translation, the proteasome, metabolism, and virulence (80). Here, proteomic profiling uncovered a novel drug-repurposing strategy using the FDA-approved anticancer drug bortezomib to treat cryptococcal infections by interfering with the ubiquitin-proteasome pathway, which modified capsule production. Alternatively, the influence of Pka1 on the secretome of C. neoformans was also investigated using bottom-up proteomics and defined the first biomarkers of cryptococcal infection (81). The secretome profile identified 61 proteins, of which, five extracellular proteins were regulated by Pka1, including Cig1, a protein previously demonstrated to be associated with virulence (82). Multiple-reaction monitoring (a targeted proteomics strategy) was subsequently employed to exploit the secretome data to quantify the secreted proteins of interest in macrophage lysates and from the blood and BAL fluid of infected mice as markers of infection.

### Biomarker identification.

The emerging systemic granulomatous disease paracoccidioidomycosis (PCM) manifests from *Paracoccidioides* spp., facultative intracellular pathogens of alveolar macrophages. PCM is endemic to Latin America, but there are weak epidemiological data as a result of noncompulsory reporting as well as high rates of false-positive results. In an attempt to identify possible biomarkers to increase the capacity of laboratory species-specific diagnosis, an immunoproteomics approach was exercised in a complementary study (83). Here, the extracellular extracts from four species within the *Paracoccidioides* complex were applied for immunization of BALB/c mice in combination with immunoprecipitation to identify reactive fungal extracellular antigens. In total, 79 exoantigens were identified across the complex, of which two antigens were specific to *Paracoccidioides lutzii*. In addition, 44 epitopes unique to the *Paracoccidioides* complex along with 12 antigenic sequences exclusive for species differentiation have potential use in diagnostic and epidemiological monitoring.

### Defining virulence.

The combination of two proteomic platforms for profiling fungal response to signaling pathway modulation describes an advanced methodology for translational use with emerging fungal pathogens to gather insight into new treatment options and biomarker discovery. For example, C. gattii features a similar infection process to that of other *Cryptococcus* spp.; however, its intense proliferative ability dictates a more aggressive progression of infection, rendering it hard to manage (84). Recently, a global survey of protein changes observed in rat lungs infected by a virulent or avirulent strain of C. gattii was mapped using bottom-up proteomics (85). In this investigation, 2,097 host proteins were identified, in which 77 were differentially produced, including the upregulation of major glycolytic enzymes and glucose transporters coordinated with reduced production of tricarboxylic acid (TCA) cycle proteins. This metabolic shift in the host cells away from oxidative phosphorylation and toward glycolytic mechanisms of ATP generation supports the energetic replication of the pathogen by opening up supply routes to nutrients and metabolites. Activation of the glycolytic pathway and downregulation of the TCA cycle was confirmed by measurement of lactate and lactate dehydrogenase in C. gattii-infected lungs and lung fibroblast cells. This metabolic status of glycolysis activation and lactate accumulation reflects the Warburg effect of proliferating cancer cells (86). Previously, the roles of Warburg metabolism in fungal pathogen recognition and host immune protection were highlighted in C. albicans through exploitation of glucose homeostasis as a mechanism of triggering rapid macrophage death (87). In a murine candidemia model, glucose supplementation improved host outcome, suggesting that immunometabolic interactions are a potential avenue toward future antifungal therapeutics.

### Immunomodulatory profiling.

Surface-exposed and secreted fungal proteins have recently sparked interest due to their crucial roles in host-fungal interactions and easily accessible location for novel druggable targets, biomarkers, and vaccines (88). This interest was demonstrated by the application of cell surface “trypsin shaving” combined with bottom-up proteomics on C. glabrata, Candida parapsilosis, and Candida tropicalis (89). The phenomenon of “moonlighting” proteins occurred under infection-mimicking conditions for all *Candida* spp., and three common atypical cell wall proteins were identified in all species, including pyruvate decarboxylase, enolase, and glyceraldehyde-3-phosphate dehydrogenase. Moonlighting proteins are a recently discovered virulence factor for fungal pathogens, featuring the transfer of cytoplasmic proteins primarily involved in intracellular metabolic processes to the extracellular space for involvement in unrelated, often virulence-associated, functions. In a downstream analysis by this research group, the extracellular vesicular proteome of these non-*albicans Candida* spp. was characterized, in which a large portion of identified proteins similarly were classified as moonlighting proteins and understandably featured a large overlap with proteins identified on the cell surface (90). Among the moonlighting proteins identified here, some have immunogenic properties and roles in adhesion to human extracellular matrix proteins (e.g., fibronectin, vitronectin, and laminin). Furthermore, the C. albicans enolase (Eno1) ortholog has demonstrated high abundance as an immunodominant antigen in patients featuring invasive candidiasis (91, 92). Therefore, there is potential to utilize these immunogenic proteins identified on the fungal cell wall or in vesicles as candidates for biomarkers and vaccines for the treatment of candidiasis.

Another example of extracellular profiling for the identification of possible therapeutics was explored to combat the increasing emergence of drug-resistant strains and the unprecedented aspergillosis mortality rate (93). Here, a modern systems biology approach incorporated publicly available large-scale data sets generated from previous proteomic experiments with sophisticated bioinformatic prediction software to identify potential therapeutic targets within the secretome and cell membranes of *Aspergillus* spp. This computational pipeline identifies a comprehensive archive of secreted and cell membrane proteins categorized based on clinical importance. This *in silico* organization emphasizes the antigenicity, homology, and druggability of the extracellular proteins of interest. Taken together, MS-based proteomics offers diverse and adaptable technologies to profile changes in protein abundance from fungal pathogens to discover uncharacterized virulence factors, new treatment options, and biomarkers for diagnostic and prognostic purposes. Although the application of proteomics to define fungal pathogenesis is a relatively new field of study, the data gleaned to date on well-characterized species can provide new investigative leads for emerging fungal pathogens.

## METABOLOMICS

Metabolomics profiles networks of small low-molecular-weight metabolites as the culmination of cellular processes representing the entire physiology of genomics, transcriptomics, and proteomics within an organism or cell. Chromatography-based MS is a critical analytic tool for establishing comprehensive metabolic profiles, chemical signaling, and underlying regulatory mechanisms, and for comparing the characteristics of metabolic profiles of different species (94–96). For example, secondary metabolites (SecMets) and biosynthetic gene clusters (BGCs) commonly produced by filamentous fungi are of high-interest due to their multivariate capabilities in pharmaceutical and agricultural research as well as in fungal pathogenesis, including host colonization and interactions with other microbes (97).

### Biomarker discovery.

Recently, gas chromatography-mass spectrometry (GC-MS) was used to compare the characteristics of metabolic profiles of different fungal species for biomarker discovery (98). Using precise GC-MS conditions, the metabolic footprint of lung epithelial cells infected with C. neoformans revealed putative disease progression markers at the initial colonization and early infection stages (99). This temporal analysis identified metabolite characteristics that adapt over coincubation periods as well as a novel putative biomarker, pantothenic acid, with the potential to distinguish C. neoformans infection. Pantothenic acid, a precursor of coenzyme A, is an established quorum sensing molecule in C. neoformans and has been implicated in virulence factors, including melanization and titan cell formation (100, 101). Metabolic foot printing has the potential for the noninvasive screening of patient samples and faster diagnosis with noncomplex, rapid, and reproducible procedures.

### Growth stage distinction and temporal regulation.

An alternative technique of untargeted liquid-chromatography MS (LC-MS) elucidated temporal metabolome changes of the distinct lifestyle of the hemibiotrophic pathogen Colletotrichum sublineola (95)*. Colletotrichum* spp. are a serious threat to global crop security as detrimental phytopathogens capable of multistage infection pathology involving biotrophic host penetration and necrotrophic nutrient acquisition (102). Dynamic changes in *C. sublineola* metabolism during growth were indicated by time-related groups of exo- and endo-“metabolite space” under glucose, arabinose, and rhamnose carbon source conditions as revealed by principal-component analysis (PCA) models and chemometrical characterization. The distinct stages of growth determined were early adaptation during the biotrophic stage, transition, and stationary necrotrophic phases. *C. sublineola* demonstrates an upregulation of time-associated metabolites, such as phytotoxins, independent of the carbon source during the biotrophic stage, and necrotrophic stage carbon source-dependent metabolites. Furthermore, the potential signalling pathways involved in fungal structure invasion, signals from the plant host contributing to growth transition, and novel discovery of *C. sublineola*’s ability to effectively use different carbon sources *in planta* lay the groundwork for future studies.

### Antifungal resistance.

To gather a global understanding of metabolites involved in resistance to antifungal treatment, a comparative metabolomic study of sensitive and drug-resistant C. albicans strains combined GC-MS methods, such as ultrahigh-performance liquid chromatography coupled with quadrupole time of flight MS (UHPLCQ-TOF/MS) and hydrophilic interaction LC-MS for phospholipid-specific metabolism (103). The MS-based methodologies identified a plethora of biomarkers related to drug stress and resistance levels with a trend toward implications in the metabolism of amino acids, sphingolipids, and phospholipids. The high-throughput resolution of this metabolomic study significantly increased the understanding of drug resistance evolution in C. albicans and offers a complementary approach to other omics-antifungal resistance analyses.

### Characterization of effector molecules.

The characterization of metabolites, or effector molecules, from complex biological systems provides an alternative perspective to the upstream commands originating from the genome, transcriptome, and proteome. A dominant virulence strategy in *Candida* spp., specifically, C. albicans, is to morphologically adapt via the production of phenotype-switching metabolites to generate true hyphae. The novel MDR C. auris lacks the ability to form true hyphae but instead forms pseudohyphae (104, 105). GC-MS analysis highlighted a different metabolic profile of C. auris compared to that of C. albicans under hypha-forming conditions, most significant was the production of hypha-inhibiting metabolites and biofilm-forming molecules such as tyrosol (96). The C. auris metabolites produced suggest a profile capable of host infection, including secreted fatty acids to impair the natural host response to eliminate microorganisms, propanoic acid to exert immune-suppressive activity, and a pyrazine derivative involved in host-microbe colonization. Overall, chromatography-based MS data support the development of a tool to sensitively detect fluctuations within complex cascades involved in both fungal infection and host defense mechanisms as an approach for disease diagnosis and antifungal development.

## MULTI-OMICS

**FIG 2:**
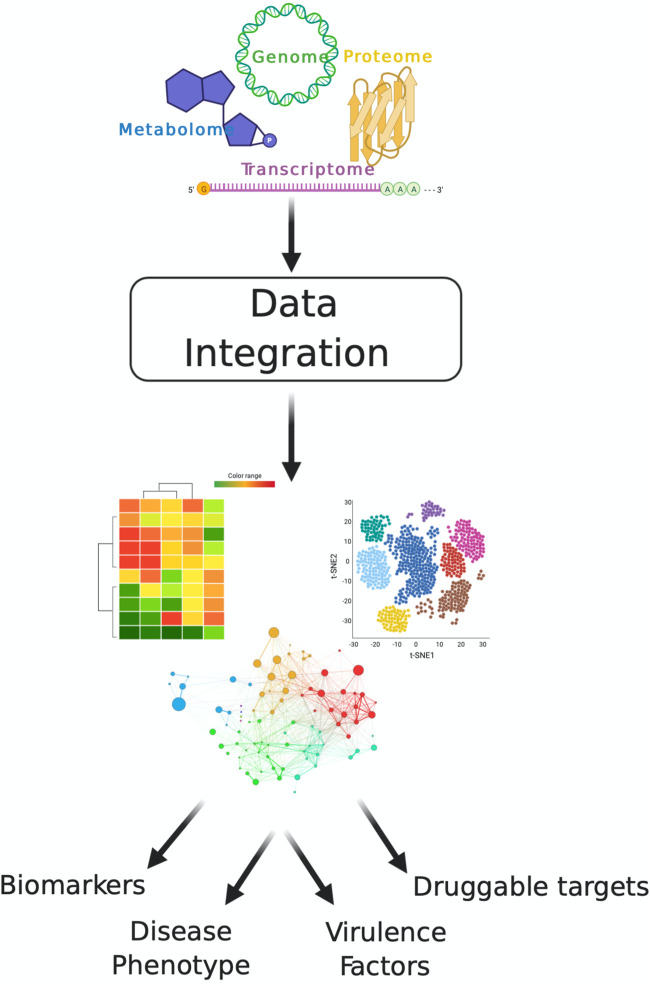
Multi-omics data integration. Multi-omics combines data from multiple platforms for comprehensive and in-depth profiling of biological processes. A critical parameter for maximizing the applicability of multi-omics profiling is data integration, which encompasses bioinformatic tools, pipelines, and databases. Data integration of multi-omics strategies promotes the development of biomarkers, definition of disease phenotypes, characterization of virulence factors, and discovery of druggable targets among other potential outcomes.

The scientific community’s adoption of individual omics technologies initially offered novel insight into the composition and structure of biological interactions within an organism. However, the rising trends of available and affordable high-throughput technologies confer the additive power of multilevel data analyses and drive the integration of multiple omics platforms to obtain a more cohesive holistic view (Fig. 2) (106). This integrated systems-level approach incorporates single omics data to unite the phenotype and the genotype. Furthermore, to assess the overwhelming flow of information, advanced bioinformatics strategies are required to assimilate the biological system comprehensively (107, 108). This significant progress allows an unbiased investigation from subtle to prominent biological interactions with predictive accuracy and revolutionizes the long-term field of mycology.

### Pathogenesis.

Recent studies combining omics technologies feature a transparent perception of the specific biological system being analyzed. For instance, the integration of comparative genomic and RNA-seq dual-organism transcriptomic analyses of *Rhizopus* and *Mucor* strains within human airway epithelial cells revealed fungal and host molecular pathways contributing to pathogenesis (109). Mucormycosis is a dangerous opportunistic invasive human infection caused by Mucorales fungi, sporadically capable of infecting healthy individuals following the events of natural disasters, causing outbreaks of cutaneous necrotizing soft tissue mucormycosis (110, 111). The comparison of 41 annotated genomes disclosed a unique concentration of CotH invasins across Mucorales fungi; the presence and gene copy number of CotH is associated with species-specific pathogenicity. Additionally, CotH exhibited higher expression in RNA-seq analyses of Rhizopus oryzae and Rhizopus delemar interactions with host cells. Furthermore, the host transcriptional response to Mucorales featured platelet-derived growth factor receptor B signaling, corroborating an angioinvasive mechanism of mucormycosis pathogenesis. In another integrated omics study, the cell wall proteome and glycome were coordinated to identify a unique mannan on the surface of the MDR C. auris capable of strongly associating with human serum IgG and mannan-binding lectin proteins (112). Recent insights into the host immune response against C. auris highlighted the requirement of opsonization via human serum for inducing cytokine production in human mononuclear cells (113). Furthermore, functional and structural assessments revealed mannans as fundamentals components for inducing a stronger cytokine response than that from C. albicans. These findings are valuable considering C. auris’ colonization of the human skin is exceptionally difficult to eradicate, binding tightly and displacing the natural skin microbiome (114).

The aggressive and emergent grapevine pathogen Lasiodiplodia theobromae, which is associated with >500 plant species and a multitude of diseases, also has a history of rare human infections, such as keratitis (115, 116). The sensitivity of this fungal agent to elevated temperatures provokes its pathogenicity; therefore, a combination of *L. theobromae* genome, transcriptome, and proteome analyses was recently performed for ambient and mammalian host temperatures (117). At 25°C, there was a substantial increase in extracellular proteins involved in plant cell wall degradation and pathogenesis, whereas a temperature increase shifted the protein and transcript profiles toward survival responses, including for the maintenance of cell wall integrity. Interestingly, the global profile of *L. theobromae* revealed a toolbox equipped for phytopathogenicity as well as for human infection, including genes and proteins involved in tissue necrosis and colonization, such as the virulence protein SSD1 expressed solely at 37°C. The emergence of this fungal pathogen is a cause of concern. As global temperatures continue to rise, the expansion of *L. theobromae*, as well as similar phytopathogenic and hemibiotrophic fungi, arms them for potential destruction in both plants and humans.

### Strain distinction.

The novelty of integrating the lipidome, proteome, and metabolome was highlighted in a comprehensive comparison of two clinical C. auris isolates to a reference C. albicans strain (118). Quantitative proteomic profiling presented a significant difference between proteins with higher abundance in the glycolysis and gluconeogenesis pathways in C. albicans, whereas C. auris exhibited enrichment of proteins in the TCA cycle and lipid metabolism. This divergence in central carbon metabolism was confirmed by the integration of proteomics data and metabolite analysis. This integration also cohesively outlined the ergosterol biosynthesis pathway, indicating a higher abundance of ergosterol synthesis enzymes in both isolates as well as a higher abundance of ergosterol itself in one of the C. auris isolates. Additionally, the lipid profile showed enriched lysophospholipids and glycerophospholipids in C. auris compared to that in C. albicans, indicating higher phospholipase activity, which was confirmed by proteomic profiling. This approach tackled numerous aspects of invasive fungal disease, antifungal resistance, and adaptation of this worrisome emerging pathogen, suggesting opportunities for the development of alternative therapeutics. Directing the effectiveness of integrated omics technologies toward understanding a novel fungal pathogen provides crucial systems-level information connecting the capacity of emergence and endemism, determinants of virulence, and the resulting disease phenotype.

## OUTLOOKS AND PERSPECTIVES

The dogma of molecular biology expresses the transfer of information from DNA to RNA to protein purposed to build a functional product; however, within a biological system, this flow of genetic information is a complex nonlinear process of interconnected pathways. Omics technology reflects the dynamic concept of biological processes and supplies a contemporary approach to mycology research for fungal pathogen management by outlining the evolution of emerging species (i.e., lineage-specific chromosomes), identifying putative biomarkers, and describing the fine-tuned interactions within infection settings. This robust output of information contains predictive power for anticipated emerging fungal pathogens in addition to precision medicine, and these concepts offer an opportunity for mitigation to avoid the future implications of infectious diseases. This ideology encourages the potential of rapid positive diagnosis followed by efficient targeting and eradication of mycoses without detrimental effects to the host.

The concept of omics and its downstream futuristic revelations are exciting; however, the real-life authentic aspects of applying these technologies in a research setting consist of significant challenges and current roadblocks. The rise of inexpensive genome sequencing led to a wave of incoming raw sequence data, resulting in unverified data sets with the assembly of annotation, redundancy, and possible contaminating DNA, overall resulting in circumstances of inconsistent data (27, 119). Therefore, a multitude of resources were generated to correct and curate sequences to ensure data quality standards and to organize the vast quantities of generated data (e.g., single genus-focused databases such as *Candida* Genome Database [CGD] and multiple fungal genomes such as FungiDB) (120–122). It is necessary to ensure that omics data are held to high-quality standards, as public data sharing is an important component of research to combat infectious diseases. For example, research interests have recently focused on correlating lateral gene transfer in fungi as a mechanism to transfer pathogenicity-related genes, explaining the unique genomic compositions of some nascent fungal species (123). Therefore, to arrive at a sound conclusion of the transmission of genetic information, it is pertinent to have accurate resources.

Simultaneous profiling of pathogen and host interactions is a powerful approach to elucidate the capacity for a fungus to cause disease; however, there are also extensive difficulties in dual-organism omics analyses. For instance, metabolomics lacks a genetic template to annotate genes, transcripts, and proteins that have unique reference sequences. This poses a challenge in a dual-organism state to precisely distinguish the fungal from the host global metabolome, as many metabolites are consistent across eukaryotic organisms. To overcome this limitation, the field of infection metabolomics is evolving to access the opportunities of metabolic biomarkers, as the sensitivity of a metabolite profile greatly reflects adaptation to the surrounding microenvironment. One solution to differentiate host-pathogen metabolites is the integration of other omics technologies to correlate metabolite abundance to gene expression or protein production (124). Despite the development and increased accessibility of high-throughput sequencing and MS technology, there is still a high cost associated with the processing and measuring of large data sets, especially for dual-organism studies. For example, to tackle a systems biology approach for fungal research, it is necessary to practice cohesive sample processing across all omics levels. Sources of omics data variation may originate within the biological specimens and heterogeneity of cell types as well as from the wide source of platforms used to extract the molecular material and generate the data. The robustness of omics technology has orchestrated higher resolution of the genetic diversity between environmental, clinical, and laboratory type strains of the same fungal species, resulting in possible expression variation in biological processes. To achieve a solid representation of gene expression programs in relation to infection and pathogenesis, it is necessary to define gene patterns across multiple relevant fungal isolates (125). Moreover, the advent of single-cell sequencing technology has provided researchers an attractive approach to deepen the understanding of cell-to-cell variation. This highly specialized method uncovers pathogen and host cell heterogeneity, which population studies incidentally masked due to the averaging of the results; single-cell analysis in dual-organism studies provides a critical component of understanding fungal infection and accelerates the development of novel therapeutics (126).

It is important to address that with large multi-omics data sets comes the requirement for consistent, robust, accurate, and reliable data analysis strategies. Fortunately, the incorporation of powerful computational and algorithmic techniques provided in publicly available platforms may be utilized to answer the biological questions in mind. However, it is necessary to have sufficient training when dealing with the increasingly sophisticated technology as well as efficient means for storing, processing, and retrieving data. In addition, the generation of large multidata sets requires significant computational power for data integration and modeling, including data storage, quality control, and statistical analysis, all of which are necessary to achieve the desired holistic understanding of an experimental system (127). Specifically, to harmonize omics data sets, it is necessary to account for appropriate metadata collection to achieve reproducible and biologically relevant interpretation of the results (128).

The impact of the infectious fungal diseases outlined here is exhibited by crop destruction, resulting in socioeconomic consequences and instability of food security as well as hardships on health care systems from difficult-to-diagnose and -treat fungal epidemic outbreaks. This review focused on diverse applications of the primary omics approaches but did not elaborate on the benefits of epigenomics, glycomics, or lipidomics due to a limited representation of emerging fungal pathogens within these research dimensions. However, we envision the universal application of many omics technologies in the future to address complicated questions about emerging fungal pathogens and increase our understanding of current mechanisms of antifungal resistance to limit, or even prevent, the selective pressure towards resistance in emerging pathogens. We highlight diverse applications of omics technologies in well-characterized pathogens to demonstrate the potential information to be harnessed for emerging pathogens. Furthermore, we present omics data from newly emerging pathogens to explore the immense potential of such platforms in combatting the impact of fungal pathogens on a global scale.
